# Health impacts with telework on workers: A scoping review before the COVID-19 pandemic

**DOI:** 10.3389/fpubh.2022.981270

**Published:** 2022-10-31

**Authors:** Yuko Furuya, Shoko Nakazawa, Kota Fukai, Masayuki Tatemichi

**Affiliations:** Department of Preventive Medicine, Tokai University School of Medicine, Isehara, Japan

**Keywords:** telework, telecommuting, work from home, teleworker health, health impacts, before COVID-19

## Abstract

**Background:**

Telework has dramatically increased due to the coronavirus disease 2019 (COVID-19) pandemic, and the health impacts related to telework have become major concerns. Some studies have shown that telework has both positive and negative impacts. However, during the pandemic, the influence of COVID-19 is too strong to estimate the health effects of telework. Therefore, this scoping review investigated a comprehensive overview of those impacts based on studies conducted before the COVID-19 pandemic.

**Methods:**

We searched keywords related to telework in five databases: PubMed, Scopus, Cumulative Index to Nursing and Allied Health Literature (CINAHL), Cochrane Library, and Ichu-Shi Web. We included articles written in English and Japanese and published from January 2009 to December 2020. One author extracted data, and four authors were paired into two groups. All authors independently conducted the first and second screening and checked the results in pairs. Any disagreements were resolved by reaching a consensus among all authors. All screening and strategies were performed with the consent of all authors.

**Results:**

Twenty-nine quantitative studies published in 12 countries were extracted. The outcomes included 10 studies on physical and lifestyle outcomes, 25 studies on stress and mental health outcomes, and 13 studies on quality-of-life and wellbeing outcomes. Telework increased sitting time in one study, and two studies showed improvement in behavior, such as reducing smoking or drinking due to telework. While six studies reported subjective stress levels improved by telework, the results for depression, anxiety, and other disorders varied across those studies, and the social or individual factors further complicated the situation.

**Conclusion:**

Telework is potentially associated with a shift to healthier lifestyles but also the potential for inverse correlation to extend sedentary time. Mental stress indicators depend on the social and individual situations, and very few intervention studies on teleworking existed prior to the COVID-19 pandemic. Our review identified a lack of intervention and comparative research on health problems with telework and revealed a need to conduct research with clear comparisons in post-COVID-19 studies.

**Systematic review registration:**

https://www.crd.york.ac.uk/prospero/display_record.php?ID=CRD42021203104, identifier: CRD42021203104.

## Introduction

The coronavirus disease 2019 (COVID-19) pandemic, caused by the severe acute respiratory syndrome coronavirus 2 (SARS-CoV-2), has changed the workstyle of individuals. Although the infection situations are different across countries and governments of various countries have implemented several measures, this pandemic has had a huge influence on people's lives, especially as it concerns their work styles. Most individuals, except the essential workers, have shifted to telework, and most workers realized that they could work without commuting to their offices (1, 2). This change in workstyle will not return to its original state even after the coronavirus epidemic ends and will become the main workstyle for post- and with COVID-19 in future.

Telework has changed drastically due to the COVID-19 pandemic, but the evolutionary circumstances differed among countries. In some western countries, telecommuting has been commonplace since the 1970s. People gradually gained interest in flexible work and working from home after the 1980s as women advanced into society (3). In addition, the telecommute concept expanded based on support for an employee with a disability, environmental protection, and business continuity plan (BCP) (3). Now, several companies and organizations have introduced the rules of telework. The European Foundation for the Improvement of Living and Working Conditions (Eurofound) and the International Labor Organization (ILO) released a joint report about working anytime and anywhere in 2017 (4). With the spread of high-speed Internet, “Telework/ICT-mobile work” can be defined as the use of ICT—such as smartphones, tablets, laptops, and desktop computers for the purpose of work outside the employer's premises (4). This report showed research results from 10 European Union (EU) countries (Belgium, Finland, France, Germany, Hungary, Italy, the Netherlands, Spain, Sweden, and the UK) and five ILO countries (Argentina, Brazil, India, Japan, and the USA). According to this report, there are more people among occasional teleworkers than among regular teleworkers in most countries and showed that the penetrating ability of telework depends on countries and occupations and ranged from 2 to 40% (4).

Previous studies have shown that telework has both positive and negative impacts (5–7). For example, the confirmed positive impacts include reduced commuting time, more work flexibility, and better work–life balance (5). However, negative impacts include long working hours and the increasing obscurity in the borderline between private life and official work (6). Also, people conflict between work and life balance, leading to increased stress affecting physical and mental health (7). To the best of our knowledge, there were no reviews of health issues related to telework published before the beginning of the COVID-19 pandemic, and many uncertainties remain regarding the positive and negative impacts of telework.

Telework is a recognized concept with several terms: telecommuting, telework, remote work, and flexible work. Allen et al. organized the concepts of telework in a table (3), and the concepts were strictly defined in different terms. According to Allen et al. (3), flexible work arrangement refers to the overall option of working beyond the standard operating days and locations. Remote work defines a form of full-time teleworker who lives and works outside the commuting area, and telework and telecommuting have set the broader concepts that include remote work from home or satellite office, and a form of work in partially or completely replace to commute.

Many studies on long-term health problems for telework during the COVID-19 pandemic are likely ongoing, but study population, work, and telework methods may not necessarily correspond to eligible targets among studies because of the diverse culture and acceptability for telework. This study aimed to systematically investigate any health impact on teleworkers. Along with the health impacts related to telework in the COVID-19 pandemic, the influences of COVID-19 are too strong to estimate the health effects of telework. Therefore, this review focused on studies published up to the early days of COVID-19 pandemic. The purpose of this review is to identify relevant health issues related to telework and what is missing in existing research and to add fundamental information for future systematic reviews.

## Materials and methods

### Search strategy

We chose a method of scoping review to search a wide range of literature about telework and health. Two research questions (RQ) were developed: (1) Did telework affect workers' health? and (2) what kind of health impact was associated with telework? Health impacts included any kind of condition associated with physical and mental health. We searched five databases related to occupational health impacts: PubMed/MEDLINE, Scopus, Cumulative Index to Nursing and Allied Health Literature (CINAHL), Cochrane Library, and Ichu-Shi Web. Searched articles were limited to English and Japanese and to those published from January 2009 to December 2020. During the review process, careful consideration was given to exclude studies conducted after the COVID-19 pandemic. The reason for considering 2009 is that this was the year of change in world labor due to the public emergence of Wi-Fi, cloud service, and 2008 global recession (Lehman shock) (8, 9). This search process was carried out by two authors (YF and KF).

Keywords used in the search included “telework,” “remote work,” “work at home,” “work from home,” “telecommuting,” “work-home,” “teleworking,” and “health.” The authors agreed on search keywords and strategy, and study designs and article types were undesignated, but commentaries were excluded. We removed overlapping reports among the publications extracted from each database. This review was based on PRISMA extension for Scoping Reviews (PRISMA-ScR) (10).

### Selection criteria

The extracted studies were required to focus on health impacts due to telework, including mental and physical health. First, studies that did not fulfill selection criteria were excluded based on the information in the title and abstracts as primary screening independently among four authors. They verified the results in pairs and retained studies that could not be determined in primary screening for a secondary screening. Next, full texts were screened by whether they matched the RQ during the secondary screening. As our criteria for the selection of this study, for physical health, we conceptualized conditions that could be considered as a disease by being examined and diagnosed, as for mental health, we targeted conditions that could be diagnosed as mental health diseases as well. Studies were included when workers were the study subjects but excluded when all subjects were students, homemakers, and nonemployee. Studies with an outcome related to productivity were also excluded. Two authors conducted the secondary screening in pairs, and any disagreements were resolved by reaching a consensus among all authors. The secondary screening results, as well as reasons for excluding some articles, were noted. The selection criteria were the following: (1) participants were wholly or partly workers, (2) all or parts of study outcomes answered the RQs, (3) exception of commentary, review articles, and qualitative studies, and (4) articles presented in English and Japanese languages. In cases where the broad concept was included in the exposure factor, yet no actual telework status was suspected based on the researcher's review, we excluded those studies under the criterion that they did not meet the RQ by the consensus of all researchers. This study was registered in the PROSPERO with registration no. CRD42021203104.

## Results

**Figure 1:**
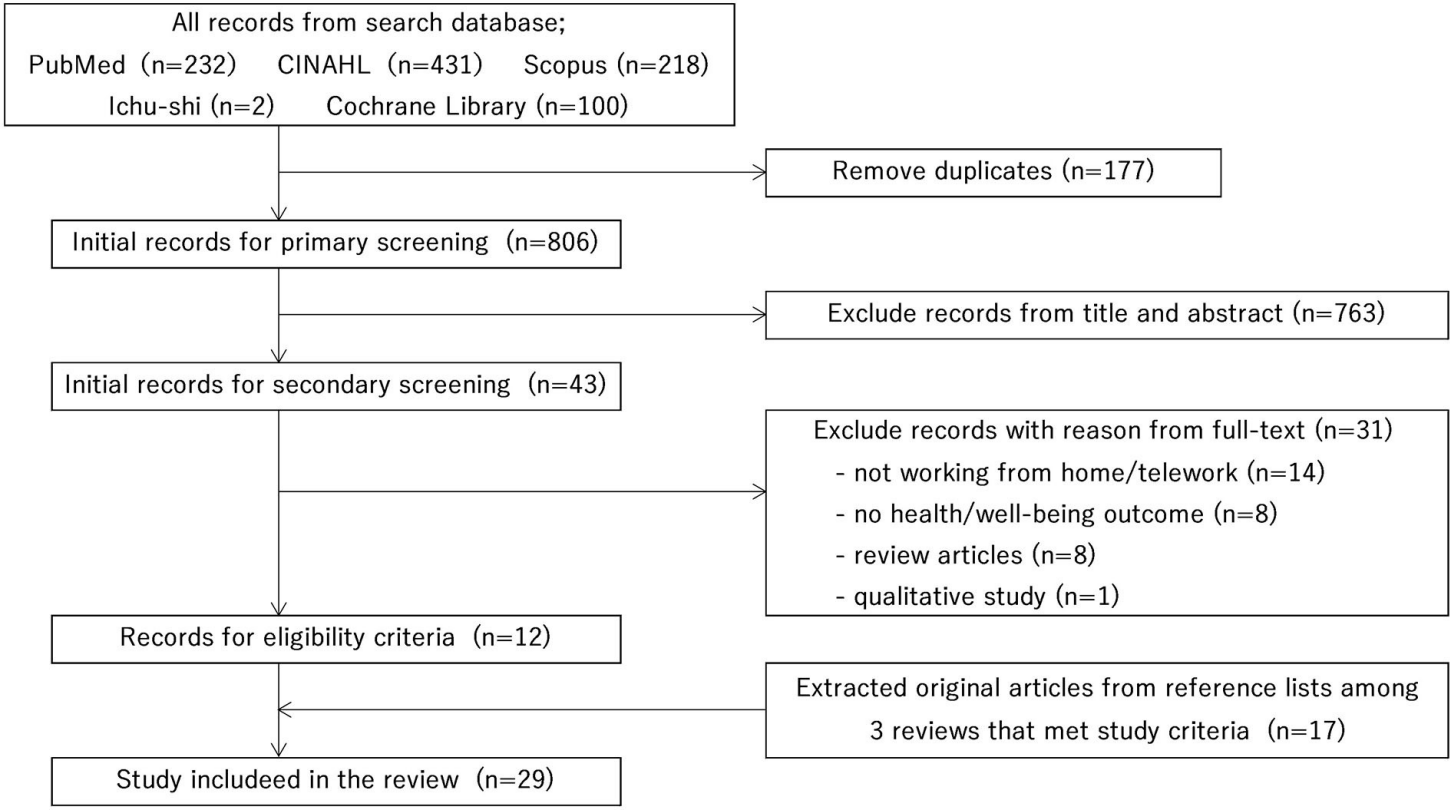
PRISMA flow diagram of the search process.

The search process is shown in a PRISMA flow diagram (Figure 1). Because of the database search, 983 records were extracted. After excluding 177 duplicate records, 806 were subjected to primary screening by title and abstract, and 43 were subjected to secondary screening by full text. Next, we excluded 31 records because the articles included subjects who were not teleworking (*n* = 14) or had no health/wellbeing outcomes (*n* = 8). Also, we excluded review articles (*n* = 8) and qualitative studies (*n* = 1). Therefore, we included 12 records. During the secondary screening, we identified three review articles with similar objectives and outcomes of this study among the 43 records. Therefore, we selected 17 eligible records among the reference lists of these reviews. We reviewed a total of 29 records (11–39).

Table 1 summarizes eligible articles included in this scoping review. Overall candidates for full screening were 29 quantitative studies (23 cross-sectional studies, four cohort studies, and two non-randomized intervention studies). These records were published from 12 countries, and most of the records (11 in total) were published from the United States. Others were five from the Netherlands, two from Australia, Belgium, and Spain, and one from Germany, South Africa, Estonia, New Zealand, Hong Kong, India, and Japan. There were 12 records published in 2020, and the remaining records were published from 2009 to 2019. Overall, 10, 25, and 13 articles were related to physical and lifestyle outcomes, stress and mental health outcomes, and quality-of-life and wellbeing outcomes, respectively.

**Table 1:** Study summary of selected articles (*n* = 29).

**No**	**Author (year) country**	**Study design**	**Participants/Telework definition (where available)**	**Health measures**	**Findings**
1	Hornung and Glaser. Germany (11)	Cross-sectional study	−1,008 employees of the German public administration who are on a telework 1–4 per week (27.5% female) - Working from home (WFH) proportion (0–100%)	A well-validated scale for quality of life by the World Health Organization (6 items)	- Home-based telecommuting had benefits for satisfaction and quality of life. - Quality of life among men were significant in contrast to that observed in women.
2	Golden. USA (12)	Cross-sectional study	−316 technology professionals in a large computing company (71% male) - WFH proportion (3–100%)	5-item scales for work exhaustion from subscale of the General Burnout Questionnaire	- More extensive telework was associated with work exhaustion.
3	Sardeshmukh et al. Australia (13)	Cross-sectional study	−417 full-time workers from a large supply chain management company (121 women) - Remote work time (8–40 h/week)	8 items scales for exhaustion developed by Maslach and Jackson	- Telework was negatively associated with exhaustion.
4	ten Brummelhuis et al. Netherlands (14)	Cross-sectional study	−110 employees from a large telecom company (48 women) - Participation to New Ways of Working (NWW): Flexible time and place using new media technologies	9-item version of the Utrecht Work Engagement Scale (UWES) and 5-item scale for exhaustion of the Utrecht Burnout Scale (UBOS)	- New Ways of Working (NWW) was positively correlated with daily work engagement and reduced exhaustion. - NWW have the possibility to improve work engagement, but could easily be changed by communication.
5	Tustin. South Africa (15)	Cross-sectional study	- Home- and office-based academics, managers of academic department, and students (154 academics, 156 students) - Home- or office-based	46 predetermined statements for telecommuting impacts (work-based and emotional)	- Telecommuting has improved quality of life, reduced stress, and less fatigue with physical and emotional.
6	Arvola and Kristjuhan. Estonia (16)	Cross-sectional study	−259 academic staff working in the Tallinn University of Technology - Remote work time (1–20, 20 h per week)	3 point scale of health complaints (hypertension, stress, and tired eyes)	−49% respondents perceived lower or rather lower stress than working at the office. - Telework did not significant increase in tired eyes complaints. - Non-telework increased complaints of stress and blood pressure.
7	Henke et al. USA (17)	Cohort study	−3,703 employees aged 18–64 working in Prudential Financial from 2010 to 2011 (62% female) - Remote work time (1–8, 9–32, 33–72, 73 h per week)	8 risk indicators; obesity, depression, stress, Tobacco use, alcohol abuse, poor nutrition, physical inactivity, and Edington score (overall risk)	- Working from home did not significantly reduce the trend for alcohol abuse, physical inactivity, poor nutrition, stress, tobacco use, obesity, and Edington score. - Younger age group had risk for depression, alcohol abuse, and poor nutrition.
					- Older age group had risk for obesity. - Female had risk for depression and stress. - Male had an increased risk for alcohol abuse, poor nutrition, and tobacco use. - The U-shaped relationship between intensity of telecommuting and health risk were depression, poor nutrition, physical inactivity and obesity. - The risk had decreased for alcohol abuse, tobacco use, and Edington score with increasing intensity.
8	Anderson et al. USA (18)	Cross-sectional study	−102 employees who teleworked at least once per period in a large US federal agency (50% female)	10 items from the job-related affective well-being (5 items for positive affective well-being (PAWB) and 5 items for negative affective well-being (NAWB))	- Telework increased experiences of PAWB and decreased experience of NAWB. - More openness and strong social connections are likely to feel positive emotions and less stress during telework.
9	Shepherd-Banigan et al. USA (19)	Cross-sectional study	−570 working women with 6–24 month children from the dataset among the National Institute of Children Health and Human Development study in 1991–1993 (specified female) - WFH time (1–8, 9–16, 17–24, 25–32, 32 h per week)	The Center for Epidemiologic Studies Depression Scale (CES-D) scores at 1, 6, 15, 24 months and the Job Role Quality Scale (21 item) at 15, 24 months	- Moving to work from home decreased depression score. - Changing telework hours per week, work schedule, and Schedule flexibility were not associated with depression score.
10	Bentley et al. New Zealand (20)	Cross-sectional study	−804 teleworkers from 28 organizations (47% female) - Remote work time (1–7, 8 h per week)	12-item General Health Questionnaire (GHQ-12) for psychological strain	- Organizational and manager supports were associated with reducing to psychological strain.
11	Nijp et al. Netherlands (21)	Interventional study	−1,443 employees from a large financial and insurance company in Dutch (case 1232, control 210, 63.9% male) - Participation to NWW	3 items from the fatigue assessment scale, 1 item for stress, and 1 item for self-reported health	- Self-reported health was decreased for teleworkers. - There was no consistent pattern of fatigue and stress.
12	Suh and Lee. Hong Kong (22)	Cross-sectional study	−258 teleworkers from two global IT companies (111 women) - Intensity of teleworking (high/ low)	Various adapted validated scales (this table, 3 items for strain)	- Low IoT teleworkers have more vulnerability to technostress than high IoT teleworkers. - Work overload, invasion of privacy, and role ambiguity are the main sources of teleworkers' strain.
13	Elst et al. Belgium (23)	Cross-sectional study	−878 employees of the Belgium branch of a telecommunication company (83% male) - WFH frequency (<1, 1, 2, 3, 4, 5 days per week)	5 emotional exhaustion and 4 cynicism items from the Utrecht Burnout Scale (UBOS-A), 9-item Utrecht Work Engagement Scale, and cognitive stress scale from the Copenhagen Psychosocial Questionnaire (COPSOQ)	- Extent of telecommuting was not directly related to work-related well-being, but indirectly related through social supports. - Extent of telecommuting was not significantly associated with burnout (emotional exhaustion, cynicism), work engagement, and cognitive stress.
14	Windeler et al. USA (24)	Study 1: cohort study (4 months follow) study 2: cross-sectional study	- Study 1: 51 participants from IT services in a financial services firm Study 2: 258 participants employed full-time and over age of 18 registered the Prolific Academic (40% female) - Remote work frequency (days per week)	4-item scale from the Maslach Burnout Inventory for work exhaustion	- Part time telework reduced interpersonal interaction and work exhaustion. (study 1) - High age men have clearly about recognizing exhaustion after telework. (study 1) - Female teleworkers were likely to feel high level work exhaustion compared to non teleworkers. (study 2)
15	Van Steenbergen et al. Netherlands (25)	Interventional study	−126 employees from a large Dutch financial services provider (65.1% female) - Participation to NWW	5 items of the Maslach Burnout Inventory for burnout, 6 items of the shortened Utrecht Work Engagement Scale (UWES) for work engagement, and psychological capital (self-efficacy, resilience, hope, and optimism)	- Transition to new ways of working did not significantly change over time for burnout and work engagement. - Teleworkers with a high level psychological capital have low burnout and high work engagement. - New ways of working led to decrease in mental demands and workload.
16	Gerards et al. Netherlands (26)	Cross-sectional study	−656 participants employed in a wide range of sectors and occupational fields - Participation to NWW	9 version of the Utrecht work engagement scale (UWES), 2 aspects of social interaction at work, and transformational leadership (emotional and spiritual quotient)	- New ways of working have significantly positive associations for work engagement. - New way of working mediated both social interaction and transformational leadership directly.
17	Kaduk et al. USA (27)	Cross-sectional study	−758 employees in a large US information technology firms from Fortune 500 corporation - Remote work proportion (<20%, 20% per week)	Six well-being outcomes (work family conflict, job satisfaction, turnover intentions, emotional exhaustion (burnout), perceived stress, and psychological distress)	- Voluntary remote work was associated with greater job satisfaction and less stress. - Involuntary remote work was not clearly linked to work family conflict, job satisfaction, emotional exhaustion, perceived stress, and psychological distress.
18	Dhont et al. Belgium (28)	Cross-sectional study	−543 radiation oncology researchers in 45 countries - Home- or office-based	14 questions to research isolation and the Hospital Anxiety and Depression Scale (HADS)	−41% and 21% persons reported anxiety and depressive symptoms in working from home. - Anxiety was significantly negative association to research experience years.
19	Majumdar et al. India (29)	Cross-sectional study	−203 corporate sector workers performing a “9–5” work at home and 325 university undergraduate or post-graduate students - WFH	Munich Chrono-Type Questionnaire (MCTQ), the Epworth Sleepiness Scale (ESS), the Center for Epidemiological Studies-Depression Scale (CES-D), the Nordic Questionnaire, and the body part discomfort rating scale	- Hypertension and gastrointestinal disturbances increased drastically among workers. - Oversleeping and nap duration had significantly increased in students and workers. - Habits of drinking and smoking were decreased, but sleep disturbance and depression were increased in students and workers.
20	Xiao et al. USA (30)	Cross-sectional study	−998 participants completely responding online questionnaire (56.5% female) - WFH	Physical and mental well-being; nine type soft physical issues and eight types of mental health issues	- Overall physical and mental well-being were decreased prior to work from home. - 64.8% reported new physical health issues and 73.6% reported new mental health issues since work from home. - Having at least 1 toddler was likely to increase the report of new health issues, and at least 1 infant was associated with one mental health issue. - Women and workers with less than 100k USD per year salary had more physical and mental issues than males with high salaries.
21	Rodríguez-Nogueira et al. Spain (31)	Cross-sectional study	−472 workers at two Spanish universities (60% female) - Remote work (yes, no)	The Standardized Kuorinka Modified Nordic Questionnaire (SNQ), Perceived Stress Scale (PSS), and frequency and type of doing physical activity	- The prevalence of musculoskeletal pain had reduced during teleworking. - Women had high stress but increased exercises during teleworking.
22	Pluut and Wonders. Netherlands (32)	Cross-sectional study	−877 workers working over the past few months in the Netherlands among 18 years or older (64% female)	Blurring of work-life boundaries (three items), emotional exhaustion (three items), happiness (single item), and lifestyle/behaviors	−26.4% participants slept worse, 39.5% less exercise, and 29.0% decreased the time to relax. - 23.1% had a positive change in healthy nutrition. - Increase in blurring of work-life boundaries conducted negative change in happiness through emotional exhaustion. - Healthy lifestyle and sleep were protective factors to inhibit blurred of work-life boundaries and emotional exhaustion for happiness.
23	Chapman and Thamrin. Australia (33)	Cohort study	−163 participants answered this survey among staff and students at five medical research institutes in Sydney (72% female) - WFH	Self-assessed mental health (one question)	−40% respondents resulted in poorer mental health in work from home. - Pajama-wearing during work from home significantly reported for mental health problems. - Work from home of participants with having children was not associated with changes in mental health.
24	Restrepo and Zeballos. USA (34)	Cross-sectional study	−1,784 individuals from 25 to 54 year-old-data among the 2017–18 Leave and Job Flexibilities Module of the American Time Use Survey (54.4% female) - WFH	Time difference about six activities (market work, personal care, leisure, sleeping, food production, and eating and drinking at home) spent between work from home or worksite work	- Leisure and sleeping significantly increased time in work from home. - Working from home led to spending more time for both the production and consumption of food at home.
25	McDowell et al. USA (35)	Cross-sectional study	−2,303 adult employees being employed before pandemic from The COVID-19 and Wellbeing (Cov-Well) Study (66% female) - WFH	Sitting time, screen time, and METs of physical activity with employment change	- Changing physical activity was not significant to change to work form home. - Sitting time and screen time increased for workers who were changing to work from home.
26	Giménez-Nadal et al. Spain (36)	Cross-sectional study	−2,471 employees aged of 16–65 without commute data from the American Time Use Survey (1106 women) - WFH proportion	5 items for well-being questionnaire from the American Time Use Survey (ATUS)	- Male teleworkers had lower level of sadness, stress, tiredness, and pain compared with male commute workers. - Female teleworkers have significantly felt a high level of happiness.
27	Kazekami. Japan (37)	Cohort study	−9,200 regular employees aged 60 years and younger from the Japanese Panel Study of Employment Dynamics - Remote work time	Each 1 item for stress, daily exhaustion, and happiness	- Male teleworkers tended to feel stress and happiness, but female did not, significantly.
28	Kim et al. USA (38)	Cross-sectional study	−6945 U.S. adults from the quality of worklife survey (3,599 women) - Workplace flexibility	Each 1 item for Job stress and daily fatigue	- Between work frequency at home and daily fatigue were not significant related. - Working at home regularly had low levels of job stress and daily fatigue. - Teleworkers who worked at home for reasons to catch up with their work experienced higher levels of job stress and daily fatigue.
29	Song and Gao. USA (39)	Cross-sectional study	−3,962 full-time and employees aged 18–65 from American Time Use Survey Well-Being Modules - WFH	6 item scales for subjective well-being (happiness, pain, sadness, stress, tiredness, and meaningfulness)	- Telework on weekdays or weekends/holidays was always associated with higher levels of stress compared to working in the workplace. - Bringing work home on weekdays increased pain and sadness, and reduced happiness for fathers. - For mothers, bringing work home on weekdays were more tired and less happy, but less stressful. - Female teleworkers with childless felt more stressed when teleworking on weekends/holidays.

### Physical and lifestyle outcomes

Table 2 shows the findings of physical and lifestyle outcomes. Physical outcomes included overall physical health issues, hypertension, musculoskeletal pain, gastrointestinal disturbances, complaints related to tired eyes, poor nutrition and obesity, physical activity, physical inactivity and sitting time, sleeping time, alcohol use, and tobacco use. One record reporting overall physical health issues revealed that telework was related to some previously unencountered health problems. One reported a worsened condition from the two available records on hypertension, while the other documented an improved situation than non-teleworkers. Three records were related to pain complaints wherein two of the records showed lower pain levels or prevalence, and the third showed that pain increased, especially in fathers. Increased gastrointestinal disturbances were documented in one report, while two reports showed an increase in screen viewing time and tired eyes complaints. Three records indicated poor nutrition and obesity. The older age group had a risk of obesity, and the younger age group and/or men had an increased risk of poor nutrition. Four records investigated physical activity, physical inactivity, and sitting time. These records on physical activity showed positive, negative, or no associations. However, one record reported that sitting times consistently increased. Of the three reports that investigated sleep issues, two showed an increase in sleeping time, but one displayed a decreasing trend. Two reports showed that alcohol and tobacco use decreased, and this tendency was positively associated with the intensity of telework.

**Table 2:** Summary of physical and lifestyle outcomes (*n* = 10).

**No**	**First author (year)**	**Overall physical health issues**	**Hypertension**	**Musculoskeletal pain**	**Gastrointestinal disturbances**	**Tired eyes complaints**	**Screen time**	**Poor nutrition**	**Obesity**	**Physical activity**	**Physical inactivity**	**Sitting time**	**Sleeping time**	**Alcohol use**	**Tobacco use**
6	Arvola (16)		↑(non-teleworker)			→									
7	Henke (17)							↑(younger group and men) U shape†a	↑(older group)		U shape†a			↑(younger group) ↓†a	↓†a
19	Majumdar (29)		↑		↑								↑	↓	↓
20	Xiao (30)	↑													
21	Rodríguez-Nogueira (31)			↓						↑					
22	Pluut (32)							↓		↓			↓		
24	Restrepo (34)							†b	†b				↑		
25	McDowell (35)						↑			→		↑			
26	Giménez-Nadal (36)			↓(men)											
29	Song (39)			↑(fathers as bring work home)											

↑ means an increase or a rise.

→ means to be flat or no change.

↓ means a decrease or decline.

†a, the association between intensity of telecommuting and risk.

†b, time for both production and consumption of food at home has increased.

### Mental and stress outcomes

Table 3 shows the findings of mental outcomes. Mental outcomes were reported as mental health issues, stress, fatigue and exhaustion, psychological strain, burnout, depression, anxiety, sadness, and relaxation. Findings on specific stress indicators, such as depression, anxiety, and fatigue, were complex. There were 12 reports on specific subjective stress indicators. Six reports indicated inverse trends, while five studies showed an increase and one showed no changes. The details were the following: Telework lowered subjective stress in men but elevated stress in women. Holiday and weekend telework for women and telework as overwork increased stress. These reports showed that voluntary teleworkers with social connections had adverse stress but teleworkers with low Internet of things (IoT) literacy were fragile for technostress. Eleven records were related to fatigue and exhaustion. Six records showed that telework lessened fatigue, two indicated increased fatigue, and two showed no changes. These records indicated that men had less fatigue, older-aged men had strong fatigue, and women tended to have a high level of fatigue. Mothers bringing overtime work at home on weekends showed exhaustion. However, these findings have various associations with telework intensity. All two records studied on overall mental health issues exhibited a worsened tendency.

**Table 3:** Summary of mental health and stress outcomes (*n* = 25).

**No**	**First author (year)**	**Overall mental health issues**	**Stress**	**Fatigue and exhaustion**	**Psychological strain**	**Burnout**	**Depression**	**Alcohol addiction**	**Anxiety**	**Sadness**	**Relaxing or relax time**
2	Golden (12)			↑†a							
3	Sardeshmukh (13)			↓							
4	Ten Brummelhuis (14)			↓							
5	Tustin (15)		↓	↓							
6	Arvola (16)		↓								
7	Henke (17)		↑(women)				↑(younger group and women) U shape †b	↑(younger group and men) ↓†b			
8	Anderson (18)		↓†c								
9	Shepherd-Banigan (19)						↓				
10	Bentley (20)				↓†d						
12	Suh (22)		↑(low IoT worker)								
13	Elst (23)		→ †b	→							
14	Windeler (24)			↓(part-time telework) ↑(older age men and women)							
15	Van steenbergen (25)					→ ↓(high social capital worker)					
17	Kaduk (27)		↓(voluntary) → (involuntary telework)	→ (involuntary telework)	→ (involuntary telework)						
18	Dhont (28)								↑†e		
19	Majumdar (29)						↑				
20	Xiao (30)	↑(women, and †f)									
21	Rodríguez-Nogueira (31)		↑(women)								
22	Pluut (32)			†g							↓
23	Chapman (33)	†h					†h				
24	Restrepo (34)										↑
26	Giménez-Nadal (36)		↓(men)	↓(men)						↓(men)	
27	Kazekami (37)		↑(men)								
28	Kim (38)		↓	↓							
29	Song (39)		↑	↑†i	↑(fathers)					↑(fathers)	

↑ means an increase or a rise.

→ means to be flat or no change.

↓ means a decrease or decline.

†a, associated with the frequency of telework.

†b, associated between intensity of telecommuting and risk.

†c, depends on the degree of social capital that workers have.

†d, depends on the support situation of the workers' surroundings.

†e, depends on workers' work experience.

†f, workers with less than 100k USD per year salary and with having at least one infant.

†g, healthy lifestyle has been protective factors to inhibit exhaustion.

†h, pajama-wearing during telework is associated with mental health problems.

†i, mothers bringing overtime work at home in weekend have increased exhaustion.

Three records reported the issue of psychological strain. These reports showed that psychological strain was associated with work overload, invasion of privacy, and role ambiguity. Women and workers with salary <100,000 were more likely to have mental strain. However, one record on burnout did not show significant associations. Four records reported on depression. A record showed a lower depression score, while increments appeared in the others.

Interestingly, telework intensity showed U shape; younger aged group and pajama-wearing individuals during work from home had a high risk of depression. One record reported that anxiety negatively correlated with longer work experience, and two records reported sadness. Men showed a general tendency to lower the level of sadness, but a father working at home was positively associated with the level of sadness. Relaxation time was reported in two records, of which one article showed that telework allowed for easier relaxation, but one reported lesser time for relaxation.

### Wellbeing and quality-of-life (QOL) outcomes

Table 4 shows the findings of wellbeing and QOL outcomes. Wellbeing and QOL outcomes were self-reported overall health, QOL, wellbeing, happiness, and engagement. Two records focused on self-reported overall health showed negative correlations with teleworkers' health.

**Table 4:** Summary of wellbeing and quality-of-life (QOL) outcomes (*n* = 13).

**No**	**First author (year)**	**Self-reported overall health**	**Quality of life**	**Well-being**	**Happiness**	**Engagement**
1	Hornung (11)		↑(men)			
4	ten Brummelhuis (14)					↑†a
5	Tustin (15)		↑			
8	Anderson (18)			↑		
11	Nijp (21)	↓				
13	Elst (23)			†b		→
15	Van Steenbergen (25)					→ †c
16	Gerards (26)					↑
20	Xiao (30)	↓		↓		
22	Pluut (32)				†d	
26	Giménez-Nadal (36)				↑(women)	
27	Kazekami (37)				↑(men)	
29	Song (39)				↓†e	

↑ means an increase or a rise.

→ means to be flat or no change.

↓ means a decrease or decline.

†a, depends on the degree of communications.

†b, depends on social supports.

†c, depends on the degree of social capital that workers have.

†d, healthy lifestyle was protective factors for happiness.

†e, happiness decreased on weekdays for fathers and decreased on weekends for mothers.

In total, two, three, and four records reported QOL, wellbeing, and happiness, respectively. Almost all manuscripts, excluding one on wellbeing and two on happiness, reported getting better based on QOL and wellbeing. Two records on happiness recorded that those teleworking got better in happiness than non-teleworkers, while one record indicated that telework was not directly associated with happiness. The blurring of work–life boundaries led to negative change, and bringing work home on weekdays by fathers and those on weekends by mothers reduced happiness. Of the four records on engagement, two showed improvements and the other two reported no changes. Engagement tends to improve together with a high level of social capital.

## Discussion

We estimated health impacts related to telework in this review. Most studies were observational, with only two being interventional. Our analysis showed that the most common outcomes were related to mental health and stress, and the next was QOL-related outcomes. The outcomes related to physical health and symptoms were present in few settings. The articles from 2009 to 2019 included two articles on physical health outcomes, 14 articles with mental health outcomes, and eight articles with QOL-related outcomes. In contrast, the number of articles on telework health problems increased sharply in 2020. There were eight articles with physical health outcomes, 11 articles with mental health outcomes, and five articles with QOL-related outcomes.

In this review, most studies were published from the United States because the United States was the birthplace of telework, followed by the Netherlands, Finland, Sweden, etc. The EU has a Framework Agreement on Telework, and about 17% of employees in the EU engage in telework on average (4). Telework was more common among managers, professionals, clerical support, and sales workers in occupations, and women were reported to be teleworking more regularly than men (4). This report suggests that the work–family model, including gender roles, is associated with the implementation rate of telework in each country (4). These differences in the background might have different effects between teleworking and health.

### Physical health outcomes

Almost all studies on the outcomes of physical health consistently showed an inverse association in the volume of alcohol consumption and numbers of tobacco smoking (17, 29) and in the complaint of pain (31, 36), and an positive association in sitting time (35). Telework could have some role on the decrease in drinking and smoking habits. As for pain relief, factors causing pain might be related to commuting time and pain could have been relieved possibly due to reducing the commuting time.

Telework was consistently associated positively with sitting time, but interestingly, there was no consistent tendency in physical activities (17, 31, 32, 35). We suspected that workers who shifted to telework could not affirm their overall physical activity had decreased because they were doing intellectual work using IoT tools in the office. Their sitting time had already become longer before the pandemic. Recently, sedentary behavior is a major concern about several health issues, particularly resulting in death (40). Therefore, the sedentary work style will be one of the emerging topics on occupational health.

### Mental health and QOL-related outcome

Mental health and QOL-related outcomes were generally inconsistent and did not show clear results. The possible reasons considered are that the effects may differ mainly from gender and family structures. In several studies, mental health outcomes were worsened among females and/or females having children (17, 24, 30, 31, 39). Gender plays different roles in society, and the blurring of the borderline between work and life and levels of support from colleagues or superiors resulted differently, suggesting that mental health outcomes might be strictly affected by the social and/or individual situations.

Subjective stress showed a decreasing and reducing trend among articles published from 2009 to 2019 (15, 16, 18, 27). There was a report that people with low IoT literacy were more likely to feel stressed (22), and another report showed that people with high social capital have higher telework-engagement and less burnout (25). This suggests that teleworker literacy, including social capital, is related to the adaptation to telework.

Telework has spread all at once by the COVID-19 pandemic. Thus, considering the impacts on health problems of workers, especially mental health, it is difficult to assess these because the influences of the pandemic, such as behavioral restrictions, economic distress, and future uncertainties, are considered to be intricately involved. Although our review estimated qualitative studies, important issues to be evaluated and to be stratified were specified. When future studies evaluate outcomes, individual stress indicators are complex. Thus, the social and personal environment should be stratified in future studies, especially in mental health. Furthermore, to consider the health effects of telework, it may be necessary to evaluate the consistency with not only one marker but also several indicators or scales.

### Strength and limitation

Our study has several strengths. One strength of this study is ensuring objectivity by the evaluation method used. The review was conducted according to an established protocol. The primary and secondary screening processes and the risk of bias assessment were evaluated independently and examined by all authors in case of disagreement between author pairs. In addition, we also extracted articles from references that were incorporated into articles on review with similar objectives published within the study period. We were able to extract many relevant articles by incorporating these articles into this study.

However, there are some limitations. First, we could not conduct a comprehensive review of the eligible articles as we excluded documents written in languages other than English and Japanese. Second, the majority of the articles included in this study were conducted in a cross-sectional design, which does not allow us to identify any causal relationships. Future cohorts and intervention studies should be conducted to address the points discussed in this review. For example, telework tends to be used more by certain groups, such as highly educated, ICT-skilled workers (41), so the possibility of multi-biases cannot be ruled out since eligible studies were mainly from observational studies. Third, we completely cannot deny some possible influences in published studies of 2020 although we tried to avoid the impacts of the COVID-19 pandemic as much as possible and extract only the impacts of telework. Fourth, telework is not necessarily limited to telecommuting, but is a concept that encompasses all forms of working outside main offices. Our study cannot eliminate the possibility that reflected each study's outcome by the differences in their respective expressions. However, despite these limitations, few reviews have examined the health impacts of telework (42–44), and this scoping review could have importance as basic information on examining the health impacts of telework in future.

## Conclusion

Our findings indicated that telework could associate inversely with mental stress and influence a shift to healthier lifestyles, although it was positively correlated with the risk of a sedentary lifestyle. However, the associations differed among family environment, gender, age, personal literacy, including IoT, and support from others. These points are useful for occupational health practice. However, we found that the associations varied by individual attributes. Telework was more likely to have a negative association on mental health for women than for men, suggesting the possibility of an interaction effect by gender, depending on environment for child care and other factors. The associations on mental health also differed depending on the level of literacy, including IoT, and social connections. The current review could not present enough evidence to withstand meta-analysis targeting each attribute due to the lack of intervention studies. Therefore, future intervention studies are required to measure health impact with adequate collection of information on such attributes.

## Data availability statement

The original contributions presented in the study are included in the article/supplementary material, further inquiries can be directed to the corresponding authors.

## Author contributions

YF and SN wrote the first draft of the article. KF and MT supervised this manuscript. All authors prepared the inclusion and exclusion criteria, screened articles, and checked for the risk of bias in all articles. All authors contributed to the article and approved the submitted version.

## Funding

This work was supported by the Ministry of Health, Labor, and Welfare under Grant (Industrial Disease Clinical Research Grants, No. 210801-01).

## Conflict of interest

The authors declare that the research was conducted in the absence of any commercial or financial relationships that could be construed as a potential conflict of interest.

## Publisher's note

All claims expressed in this article are solely those of the authors and do not necessarily represent those of their affiliated organizations, or those of the publisher, the editors and the reviewers. Any product that may be evaluated in this article, or claim that may be made by its manufacturer, is not guaranteed or endorsed by the publisher.
